# Which patients on a gynecologic oncology service will require perioperative transfusion? A single-center retrospective cohort study

**DOI:** 10.31083/j.ceog.2021.01.2152

**Published:** 2021-02-15

**Authors:** Gregory W. Kirschen, Samantha M. Dayton, Sophia Blakey-Cheung, Michael L. Pearl

**Affiliations:** 1 Department of Gynecology and Obstetrics, Johns Hopkins Medicine, MD 21287 Baltimore, United States; 2 Department of Obstetrics, Gynecology & Reproductive Medicine, Stony Brook University Hospital, Stony Brook, 11794 NY, United States; 3 Department of Obstetrics and Gynecology, Northwell Health System, Southside Hospital, 11706 NY, United States

**Keywords:** Blood loss, Hemorrhage, Minimally-invasive surgery, Cost savings, Evidence-based practice

## Abstract

The purpose of this study was to determine which patient- or surgery-related factors are predictive of need for perioperative transfusion to avoid obtaining unnecessary pre-operative type and screens (T&S). We conducted an observational retrospective cohort study of 1200 women ≥ 18 years old undergoing gynecologic surgery for benign, possibly benign, or malignant indications on a gynecologic oncology service at a university medical center from 2009-2016. A logistic regression model was used to examine patient-related and surgery-related variables predictive of outcome of transfusion. Independent variables included patient demographics, comorbidities, and surgical indication surgical route, and surgical type. Dependent variable was transfusion outcome (T&S only, conversion to type and cross (T&C), or transfusion). Eight hundred ninety-nine (74.9%) women underwent pre-operative T&S, of which 118 (9.8%) were converted to T&C, and 80 (6.7%) received a transfusion of blood or blood products. Cancer indication, major surgery, and preoperative hematocrit less than 36% were significantly associated with need for transfusion (*P* = 0.002, *P* < 0.0001, *P* < 0.0001, respectively). Patients with a benign indication undergoing minor procedures and with normal preoperative hematocrit are least likely to require transfusion.

## 1. Introduction

Every year in the United States (US), over 100,000 new gynecological cancers are diagnosed [1]. Operative management is a mainstay of treatment for many of these cancers [2]. One significant surgical risk is blood loss given not only the inherent nature of surgery, but also the vascular nature of tumors of the female reproductive tract [3]. Gynecologic cancer surgeries require treatment with blood products in as many as 13.8% of cases, and transfusion is associated with increased risk of perioperative morbidity and mortality in this population [4].

Preoperative laboratory testing accounts for approximately $3 billion of healthcare expenditures in the US alone yearly, and is likely several fold higher globally, especially given the high overutilization of such tests both within and outside of the US [2, 5]. It is unclear whether the majority of preoperative testing in the gynecologic oncology setting is necessary and/or a meaningful use of limited healthcare resources [6].

Although women undergoing gynecologic oncology surgery are considered at relatively high risk for perioperative hemorrhage, the actual proportion of women requiring transfusion represents a small minority [4]. Consequently, many of these women receive preoperative testing that is not only costly, but also does not substantially impact their care. However, it remains unclear which women undergoing gynecologic surgery will require transfusion and thus may benefit from the preoperative T&S, which is currently the standard of care at our institution.

## 2. Materials and methods

We conducted a retrospective cohort study using a convenience sample of 1200 women undergoing surgery on a gynecologic oncology service at our academic institution from 2009-2016. This study was exempt by our institution’s Institutional Review Board (IRB; protocol # IRB2019-00629). Our institution’s IRB does not require informed consent for retrospective, de-identified data. Therefore, consent was not obtained in accordance with institutional guidelines.

Data collected included patient demographics, type of procedure (major, defined as entering the peritoneal cavity, versus minor, defined as no violation of the peritoneal cavity [7]), route of procedure (open, laparoscopic, vaginal, other (e.g. vulvar)), and indication for procedure (cancer versus possible cancer versus benign). Inclusion criteria included women aged ≥ 18 who underwent surgery on the gynecologic oncology service at our institution between January, 2009 and December, 2016. We excluded patients for whom primary outcome data were missing from the electronic medical record (EMR). While our main outcome was transfusion versus no transfusion, in some instances we further separated this outcome into the following outcomes: no further hematological testing or treatment after T&S, conversion to type and cross (T&C), and transfusion. More explicitly, the data analyzed came from patients who either had no T&S sent; only T&S sent; T&S sent and patient was cross-matched (T&C); T&S sent and T&C and patient received blood products. No patients received uncross-matched blood. The transfused group included those who received a transfusion either intraoperatively or post-operatively during the same hospitalization. For patient age analysis, we used a median split to create age categories, as described previously [8].

Of note, at our institution, preoperative T&S is not mandated. Transfusion at our institution is performed based on clinician judgement and patient clinical status, without strict transfusion criteria. Hematocrit was analyzed as a categorical variable (< 36% vs. ≥ 36% and Anemia severity grades I through III as per [9]). Data were abstracted from patients of three surgeons, two hired during the study period (2011-2013), and one who left the institution during the study period.

We used univariate analysis including: T-tests, chi-square tests, Fisher’s exact tests, and ANOVA for initial analysis based on the continuous or categorical nature of each independent variable to compare each designated factor with the outcome of transfusion status. Factors that had a significant association with transfusion in univariate analysis were identified and then subjected to a regression model. We utilized a log-binomial regression analysis to examine the outcome of transfusion (vs. no transfusion) with predictive variables of age, indication, surgery type, Hct level (< 36 vs. ≥ 36) and route of surgery (MIS vs. Open). As transfusion, which occurs at a rate of under 10% in our cohort (vs. no transfusion), was utilized as the main outcome, adjusted Relative Risk was calculated for each independent variable while controlling for each subsequent.

We performed all analyses in SAS 9.4 (SAS Institute Inc., Cary, NC). We used relative risk (RR) in lieu of odds ratio (OR) because our transfusion rate was under 10% and the primary outcome was transfusion versus no transfusion [9]. We defined statistical significance as *P* < 0.05 for all analyses using two-tailed tests of significance. All data are presented as median values unless otherwise specified.

## 3. Results

Overall, 900 (75.0%) of women underwent preoperative T&S. Of these 900, 701 (77.9%) did not undergo further blood testing (i.e. conversion to T&C) or transfusion, 118 (13.1%) were converted to a T&C, and 80 (8.9%) ultimately underwent transfusion. Of the 300 patients who did not undergo T&S, none received intraoperative or postoperative T&S, T&C, or transfusion. Transfusions were evenly split between two of the three surgeons who together provided 98.5% of the data (with the remaining 1.5% of the data coming from the third surgeon, none of whose patients received a transfusion). Patient demographics and transfusion outcome are shown in Table 1. Of the 1200 women, 1033 (87.5%) identified as white/Caucasian, 78 (6.6%) as black/African American, 19 (1.6%) as Asian, and 51 (4.3%) as other. The mean age was 55.5 (± 14.9) and the mean body mass index (BMI) was 30.2 (± 9.2). Median age and BMI of those transfused was 57 years and 28.4, respectively, while median age and BMI of those not transfused was 57 years and 28.0, respectively. Overall, before adjusting for other factors (below), higher patient age was significantly predictive of need for transfusion, considering three outcomes of T&S alone, conversion to T&C, and transfusion (*P* = 0.025) (Table 1). Analyzing the same independent variables and examining the binary outcome of transfusion versus no transfusion, we found a trend towards higher age being associated with greater need for transfusion (*P* = 0.085) (Table 2). Patient comorbidities are shown in Table 3. The only comorbidity significantly associated with transfusion was hypothyroidism (*P* = 0.011) (Table 3).

In terms of preoperative surgical indications, 681 (57.2%) had a diagnosed or suspected cancer, and 508 (42.7%) had a benign condition (Table 4). Of all surgeries, 678 (57.1%) were major (entering a major body cavity, in this case the peritoneal cavity [7]) while 510 (42.9%) were minor (no violation of the peritoneal cavity). Four hundred sixty-eight (39.3%) were performed via an open (laparotomy) approach, 113 (9.5%) were performed via laparoscopic approach, 566 (47.6%) were performed vaginally (e.g. hysteroscopy, dilation and curettage), and 43 (3.6%) were classified as other (e.g. vulvar surgery). Surgical indication was significantly predictive of need for transfusion (*P* = 0.002). Patients undergoing major surgery were significantly more likely to need transfusion as compared to those undergoing minor surgery (*P* < 0.0001). Similarly, patients undergoing open surgery were most likely to need transfusion as compared to patients undergoing surgery through other routes (*P* < 0.0001). Finally, those with lower pre-operative hematocrit (Hct) levels were more likely to need transfusion as compared to those with higher pre-operative Hct levels (*P* < 0.0001) (Table 4). Examining the degree of anemia and need for transfusion, we found that more severe anemia was associated with significantly increased transfusion rate (*P* < 0.0001; Table 5).

Finally, we wondered whether various patient- or surgery-related factors would be independently predictive of need for transfusion, controlling for other patient- or surgery-related factors (Table 6). When controlling for patient age, indication, and Hct levels, major surgery, relative to minor surgery, was associated with a 3.625-fold increased risk of transfusion (*P* = 0.003). Moreover, Hct levels < 36, relative to Hct levels ≥ 36, was associated with a 3.357-fold increased risk of transfusion controlling for patient age, surgery type, and surgical indication (*P* < 0.0001). Surgical indication was not significantly associated with need for transfusion when controlling for age, type of surgery, and pre-operative Hct. Similarly, age was not significantly associated with need for transfusion when controlling for type of surgery, surgical indication, and pre-operative Hct (Table 6). We went on to compare risk of transfusion with aggregated minimally invasive approaches (e.g. vaginal, laparoscopic, vulvar) versus open approach, and found that those undergoing an open approach were significantly more likely to receive transfusion (3.0% in aggregated minimally invasive versus 12.6% in open, *P* < 0.0001) (Table 7).

## 4. Discussion

In our cost-conscious healthcare climate, we seek to decrease expenditures while maintaining high value care. A notable recent example is the introduction of enhanced recovery after surgery (ERAS) protocols, which aim to reduce hospital lengths of stay and associated costs while optimizing recovery and return to normal life after surgery [10, 11].

With respect to preoperative testing, a single T&S costs between $75-$100 [12]. Approximately 5,000,000 obstetrical and gynecologic procedures are performed in the US annually, of which 29% (1,450,000, including 500,000 hysterectomies) are exclusively gynecologic [13]. Thus, preoperative T&S accounts for approximately $108,750,000 (at $75 each) to $145,000,000 (at $100 each) of the cost of gynecologic surgery annually in the US alone [12].

Globally, the volume of surgery has been estimated to be on the order of ~234 million major surgical procedures per year, with surgical procedures accounting for a relatively greater proportion of healthcare dollars spent per capita in high- and middle-expenditure countries versus low expenditure countries [14]. There have been international efforts to map out and eliminate cost-inefficient care in the perioperative setting, including for patients undergoing gynecologic surgery, which have demonstrated substantial unnecessary costs including inappropriate screening tests [15, 16]. Still, much of this work has investigated bundled care costs without pinpointing individual tests which may be superfluous, and little has been conducted specifically within the gynecologic oncology setting.

Here, we were motivated to determine which factors may predict need for transfusion, with the goal of eliminating unnecessary pre-operative T&S testing. This is a relevant issue in the gynecologic oncology setting in which up to 14% of patients require perioperative transfusion, as compared to 1-2% of patients undergoing surgery on a benign gynecology service [4, 17]. We found an overall transfusion rate of 6.7%. Patients who were older, who underwent more invasive operations, including hysterectomy, and who underwent surgery for a known cancer or possible cancer were more likely to need transfusion and may benefit from a pre-operative T&S. While we initially stratified our outcomes according to type and screen alone versus conversion to type and cross versus transfusion (Table 1), we believed that the most relevant outcome transfusion versus no transfusion, thus in subsequent analyses we used this binary outcome. Interestingly, we found that of the patient comorbidities examined, hypothyroidism was associated with need for transfusion (Table 3), which may be related to decreased factor VIII activity and prolonged partial thromboplastin time in this population [18]. By contrast, factors such as patients’ BMI and other comorbidities were not predictive of need for transfusion. Controlling for other surgery- and patient-related factors, major surgery was the only significant risk factor for need for transfusion. These findings will help clinicians decide whether to order a pre-operative T&S prior to gynecologic surgery. If these findings are adopted widely in appropriate patients, cost savings would be substantial. For instance, in the current study, of all the patients who received T&S, 487/508 patients with benign indications were not transfused, 500/510 patients undergoing minor surgery were not transfused, and 688/710 patients with preoperative Hct of greater than or equal to 36% were not transfused. By a conservative estimate, our institution spent $60,000 on the approximately 800 “unnecessary” T&S’s over the study period, assuming $75 per T&S. Based on these data, we anticipate implementing institution-wide changes in the near future and conducting a cost-savings analysis.

Several limitations of the present work must be discussed. Firstly, we conducted a retrospective study at a single institution. Future work should examine the question of which patients require pre-operative T&S in a prospective nature, ideally among a large group of patients in multiple, varied institutions. Secondly, in our cohort, a minority of patients requiring major surgery underwent surgery via a minimally invasive approach (laparoscopy, robotic) due to surgeon preferences at our institution during the timeframe captured. On the other hand, our preoperative testing policy is standardized across the institution and is not based on individual surgeon’s preferences. With the increasing use of minimally-invasive approaches in gynecologic oncology [19], future studies should determine whether transfusion requirements vary in a larger group of patients undergoing surgery via minimally invasive versus laparotomy approaches. It has been noted that patients undergoing robotic and laparoscopic surgery for complex operations such as cytoreduction for ovarian cancer or radical hysterectomy for cervical cancer can undergo successful surgery with low blood loss and minimal risk of transfusion [20-22]. Given the increasing use of minimally invasive surgery in the gynecologic oncology setting, it will be important to assess need for preoperative type and screen in this group of patients, which is likely lower than that of patients undergoing open procedures. Nevertheless, the open approach remains common, especially in low resource settings and across the developing world. Thus, our findings could be particularly applicable to these populations where cost savings is of great concern.

While we chose to examine a host of patient-related and surgery-related factors that could have influenced need for transfusion, other factors not directly addressed in this study might also predict likelihood for perioperative transfusion in the gynecologic oncology setting. For instance, molecular markers such as BRCA mutation status in ovarian cancer patients can be used to risk stratify patients and prognosticate various clinical endpoints including surgical procedure performed, operative time, estimated blood loss, and hospital length of stay [23, 24]. Other factors that we did not specifically examine in this study but which may be associated with risk of transfusion include perioperative neoadjuvant chemotherapy, preoperative anemia, and preoperative transfusion. WHO performance status and Cherlson Comorbidity Index are also important to consider in evaluating which patients may not tolerate significant blood loss and who are also at increased risk for perioperative transfusion. Future work should seek to incorporate these additional factors into risk models for perioperative transfusion in the gynecologic oncology setting.

Finally, it is important to bear in mind in interpreting our findings that 509 of the 900 patients (56.6%) who underwent T&S had benign findings on final pathology (Table 3). Hence, the majority of patients undergoing surgery on the gynecologic oncology service in this study interval were not cancer patients, although these rates are fairly typical [25].

## 5. Conclusions

In summary, we have shown that women undergoing surgery on a gynecologic oncology service are more likely to require transfusion if they had cancer, required a major operation, or had a lower Hct at baseline. Based on these findings, we feel it is reasonable for providers to consider forgoing routine T&S in patients who are known to have a benign condition, are undergoing a minor operation, and have normal preoperative hematocrit levels. It should be noted, however, that surgical factors such as route/approach may be more of a function of individual surgeon preference/skill, and thus future work should seek to determine whether specific diagnoses or procedures performed by laparotomy versus laparoscopy versus vaginal approach are associated with greater or lesser need for perioperative transfusion.

## Figures and Tables

**Table 1.** Patient Demographics and type & screen outcome.

	Overall (n =1200)		Not transfused (n = 701)		Converted to T & C but not transfused (n = 118)		Transfused (n = 80)		*P*-Value*
**Characteristic**	No.	%	No.	%	No.	%	No.	%	
**Age**									
**Mean**	55.5		55.6		58.8		58.1		
**St. Dev**	14.9		13.6		12.9		12.9		0.025
**BMI**									
**Mean**	30.2		30.0		30.8		31.1		
**St. Dev**	9.2		9.3		7.8		10.4		0.433
**Race**									
**White**	1033	87.5	614	88.9	97	82.9	72	90.0	
**Asian**	19	1.6	9	5.6	2	7.7	0	7.5	
**Black**	78	6.6	39	1.3	9	1.7	6	0.0	
**Other**	51	4.3	29	4.2	9	7.7	0	2.5	0.407
**Ethnicity**									
**Hispanic**	88	9.0	53	9.2	10	9.1	3	4.6	
**Not Hispanic**	890	91.0	521	90.8	100	90.9	63	95.5	0.442

Abbreviations: T&S, Type and Screen; T&C, Type and Cross.

* *P*-Value compares outcomes of Transfusion Status: Transfused vs. Not Transfused.

Age: t-test; BMI: Wilcoxon-Mann-Whitney test; Race: Fisher’s exact test; Ethnicitiy: Fisher’s exact test.

**Table 2.** Patient Demographics and Transfusion Status.

	Not Transfused (n= 1111)		Transfused (n = 81)		*P*-Value*
**Characteristic**	No.	%	No.	%	
**Age**					
**Mean**	55.3		58.2		
**St. Dev**	15.1		13.1		0.085
**BMI**					
**Mean**	30.1		31.3		
**St. Dev**	9.1		10.4		0.266
**Race**					
**White**	954	87.4	73	90.1	
**Asian**	71	6.5	6	7.4	
**Black**	19	1.7	0	0.0	
**Other**	47	4.3	2	2.5	0.700
**Ethnicity**					
**Hispanic**	84	9.3	3	4.5	
**Not Hispanic**	822	90.7	64	95.5	0.265

* *P*-Value compares outcomes of Transfusion Status: Transfused vs. Not Transfused. One-way ANOVAs.

**Table 3.** Patient Comorbidities and Transfusion Status.

	Not Transfused (n= 1111)		Transfused (n = 81)		*P*-Value*
**Comorbidity**	No.	%	No.	%	
**Hypertension**	386	34.7	30	37.0	0.676
**Hyperlipidemia**	186	16.7	10	12.4	0.303
**Obesity**	435	39.2	32	39.5	0.950
**Diabetes**	132	11.9	12	14.8	0.434
**Hyperthyroidism**	12	1.08	0	0.0	1.000
**Hypothyroidism**	107	9.6	15	18.5	0.011
**COPD**	40	3.6	4	4.9	0.535
**Asthma**	111	10.0	6	7.4	0.451
**OSA**	41	3.7	1	1.2	0.357
**GERD**	126	11.3	7	8.6	0.456
**Diverticulosis**	26	2.3	3	3.7	0.441
**Kidney Stones**	22	2.0	2	2.5	0.676
**ETOH Use**	553	51.2	36	45.0	0.284

Abbreviations: OSA, Obstructive Sleep Apnea; GERD, Gastroesophageal Reflux Disease; COPD, Chronic Obstructive Pulmonary Disease; ETOH, Alcohol.

* *P*-Value compares outcomes of Transfusion Status: Transfused vs. Not Transfused. Hypertension, Hyperlipidemia, Obesity, Diabetes: Chi-square test; Hyperthyroidism, COPD, Asthma, Sleep apnea, Diverticulosis, Kidney stones: Fisher’s exact test; Hypothyroidism, GERD: Chi square test.

**Table 4.** Surgery characteristics and Transfusion Status.

	Not Transfused (n= 1111)		Transfused (n = 81)		*P*-Value*
**Characteristic** **Indication**	No.	%	No.	%	
**Cancer/Possible Cancer**	621	56.1	60	74.1	
**Benign Surgery**	487	44.0	21	25.9	0.002
**Major**	607	54.8	71	87.6	
**Minor Surgery**	500	45.2	10	12.4	< 0.0001
**Route**					
**Open**	409	36.9	59	72.8	
**Laparoscopic**	109	9.8	4	4.9	
**Vaginal**	550	49.6	16	19.8	
**Other**	41	3.7	2	4.5	< 0.0001
**Hct**					
**< 36**	240	25.86	40	55.56	
**≥ 36**	688	74.14	32	44.44	< 0.0001

* *P*-Value compares outcomes of Transfusion Status: Transfused vs. Not Transfused. One-way ANOVAs.

**Table 5.** Degree of Anemia and Transfusion Status.

	Not Transfused (n= 1111)		Transfused (n = 81)		*P*-Value*
**Grade**	No.	%	No.	%	
**0**	688	74.1	32	44.4	
**1**	196	21.1	21	29.2	
**2**	38	4.1	18	25	
**3**	6	0.65	1	1.4	< 0.0001

* *P*-Value compares outcomes of Transfusion Status: Transfused vs.

Not Transfused. Grades: 0 signifies Hct > 36%, 1 signifies Hct between 30 and 36%, 2 signifies Hct between 24 and 30%, and 3 signifies Hct < 24% [9]. Chi square test.

**Table 6.** Log-Binomial Regression by Transfusion Status Outcome.

Characteristic	Relative Risk	95% Confidence Interval	*P*-Value
**Age (Continuous)**	1.002	(0.986, 1.017)	0.829
**Indication (CA or Possible CA vs. Benign)**	0.991	(0.594, 1.653)	0.972
**Surgery (Major vs. Minor)**	3.625	(1.556, 8.444)	0.003
**Hct (< 36 vs. ≥ 36)**	3.357	(2.137, 5.274)	< 0.0001
**Route (MIS vs. Open)**	0.612	(0.3289, 1.1388)	0.121

Abbreviations: CA, Cancer; Hct, Hematocrit.

**Table 7.** Transfusion risk by route of surgery.

	Not Transfused (n = 1109)		Transfused (n = 81)		*P*-Value*
**Route**	No.	%	No.	%	
**Minimally invasive**	700	96.95	22	3.05	< 0.0001
**Open**	409	87.39	59	12.61	
Total*	1109	93.19	81	6.81	

* Missing data points =10. Chi-square test.

## References

[1] ACS. Cancer Facts & Figures 2018. American Cancer Society. 2018.

[2] Kushnir CL, Díaz-Montes TP. Perioperative care in gynecologic oncology. Current Opinion in Obstetrics & Gynecology. 2013; 25: 23–28. 23299091 10.1097/GCO.0b013e32835b80f5

[3] Stefansson IM, Salvesen HB, Akslen LA. Vascular proliferation is important for clinical progress of endometrial cancer. Cancer Research. 2006; 66: 3303–3309. 16540684 10.1158/0008-5472.CAN-05-1163

[4] Prescott LS, Aloia TA, Brown AJ, Taylor JS, Munsell MF, Sun CC, Perioperative blood transfusion in gynecologic oncology surgery: analysis of the national surgical quality improvement program database. Gynecologic Oncology. 2015; 136: 65–70. 25451693 10.1016/j.ygyno.2014.11.009 PMC4492800

[5] Zhi M, Ding EL, Theisen-Toupal J, Whelan J, Arnaout R. The landscape of inappropriate laboratory testing: a 15-year meta-analysis. PLoS One. 2014; 8: e78962. 10.1371/journal.pone.0078962 PMC3829815 24260139

[6] CM St. Clair, Shah M, Diver EJ, Lewin SN, Burke WM, Sun X, Adherence to evidence-based guidelines for preoperative testing in women undergoing gynecologic surgery. Obstetrics & Gynecology. 2010; 116: 694–700. 20733454 10.1097/AOG.0b013e3181ec448d

[7] Earl R Definition of major and minor surgery: a question and an answer. Annals of Surgery. 2007; 65: 799. 10.1097/00000658-191706000-00014 PMC1426523 17863736

[8] Miller SL, Celone K, DePeau K, Diamond E, Dickerson BC, Rentz D, Age-related memory impairment associated with loss of parietal deactivation but preserved hippocampal activation. Proceedings of the National Academy of Sciences. 2008; 105: 2181–2186. 10.1073/pnas.0706818105 PMC2538895 18238903

[9] Ranganathan P, Aggarwal R, Pramesh CS. Common pitfalls in statistical analysis: odds versus risk. Perspectives in Clinical Research. 2015; 6: 222–224. 26623395 10.4103/2229-3485.167092 PMC4640017

[10] Bisch SP, Wells T, Gramlich L, Faris P, Wang X, Tran DT, Enhanced Recovery after Surgery (ERAS) in gynecologic oncology: system-wide implementation and audit leads to improved value and patient outcomes. Gynecologic Oncology. 2018; 151: 117–123. 30100053 10.1016/j.ygyno.2018.08.007

[11] Varadhan KK, Neal KR, Dejong CH, Fearon KC, Ljungqvist O, Lobo DN. The enhanced recovery after surgery (ERAS) pathway for patients undergoing major elective open colorectal surgery: a meta-analysis of randomized controlled trials. Clinical Nutrition. 2010; 29: 434–440. 20116145 10.1016/j.clnu.2010.01.004

[12] Halder R, Liu R. When should a type and screen not be ordered preoperatively. Journal of Anesthesia and Clinical Research. 2013; 4: 272.

[13] Oliphant SS, Jones KA, Wang L, Bunker CH, Lowder JL. Trends over time with commonly performed obstetric and gynecologic inpatient procedures. Obstetrics and Gynecology. 2010; 116: 926–931. 20859157 10.1097/AOG.0b013e3181f38599 PMC3253706

[14] Weiser TG, Regenbogen SE, Thompson KD, Haynes AB, Lipsitz SR, Berry WR, An estimation of the global volume of surgery: a modelling strategy based on available data. The Lancet. 2008; 372: 139–144. 10.1016/S0140-6736(08)60878-8 18582931

[15] Chung F, Yuan H, Yin L, Vairavanathan S, Wong DT. Elimination of preoperative testing in ambulatory surgery. Anesthesia and Analgesia. 2009; 108: 467–475. 19151274 10.1213/ane.0b013e318176bc19

[16] Likitdee P, Lumbiganon P, Thongrong C, Kietpeerakool C, Kongwattanakul K. Appropriateness of preoperative screenings in patients undergoing elective gynecologic surgery at Srinagarind Hospital, Khon Kaen University, Thailand: an observational study. Thai Journal of Obstetrics and Gynaecology. 2017; 25: 223–231.

[17] Pandya LK, Lynch CD, Hundley AF, Nekkanti S, Hudson CO. The incidence of transfusion and associated risk factors in pelvic reconstructive surgery. American Journal of Obstetrics and Gynecology. 2017; 217: 612.e1–612.e8. 28709582 10.1016/j.ajog.2017.07.005

[18] Ford HC, Carter JM. Haemostasis in hypothyroidism. Postgraduate Medical Journal. 1990; 66: 280–284. 2201013 10.1136/pgmj.66.774.280 PMC2429394

[19] Conrad LB, Ramirez PT, Burke W, Naumann RW, Ring KL, Munsell MF, Role of minimally invasive surgery in gynecologic oncology: an updated survey of members of the society of gynecologic oncology. International Journal of Gynecologic Cancer. 2015; 25:1121–1127. 10.1097/IGC.0000000000000450 PMC4478101 25860841

[20] Gallotta V, Cicero C, Conte C, Vizzielli G, Petrillo M, Fagotti A, Robotic versus laparoscopic staging for early ovarian cancer: a case-matched control study. Journal of Minimally Invasive Gynecology. 2017; 24: 293–298. 27856387 10.1016/j.jmig.2016.11.004

[21] Gallotta V, Conte C, Federico A, Vizzielli G, Gueli Alletti S, Tortorella L, Robotic versus laparoscopic radical hysterectomy in early cervical cancer: a case matched control study. European Journal of Surgical Oncology. 2018; 44: 754–759. 29422253 10.1016/j.ejso.2018.01.092

[22] Gallotta V, Fagotti A, Fanfani F, Ferrandina G, Nero C, Costantini B, Laparoscopic surgical management of localized recurrent ovarian cancer: a single-institution experience. Surgical Endoscopy. 2014; 28: 1808–1815. 24414460 10.1007/s00464-013-3390-9

[23] Gallotta V, Bruno M, Conte C, Giudice MT, Davia F, Moro F, Salvage lymphadenectomy in recurrent ovarian cancer patients: analysis of clinical outcome and BRCA1/2 gene mutational status. European Journal of Surgical Oncology. 2020; 46: 1327–1333. 32085925 10.1016/j.ejso.2020.01.035

[24] Gallotta V, Conte C, D’Indinosante M, Capoluongo E, Minucci A, De Rose AM, Prognostic factors value of germline and somatic brca in patients undergoing surgery for recurrent ovarian cancer with liver metastases. European Journal of Surgical Oncology. 2019; 45: 2096–2102. 31227342 10.1016/j.ejso.2019.06.023

[25] Savelli L, De Iaco P, Santini D, Rosati F, Ghi T, Pignotti E, Histopathologic features and risk factors for benignity, hyperplasia, and cancer in endometrial polyps. American Journal of Obstetrics and Gynecology. 2003; 188: 927–931. 12712087 10.1067/mob.2003.247

